# 638. Broader Empiric Therapy or Rapid Diagnostics for Hospital-onset Sepsis? A Retrospective Cohort Study of Antibiotic Resistance Prevalence and Empiric Antibiotic Adequacy

**DOI:** 10.1093/ofid/ofaf695.202

**Published:** 2026-01-11

**Authors:** Morgan Walker, Scott Sorongon, Jonathan Baghdadi, Katherine E Goodman, Sarah Warner, Emily E Ricotta, Anthony Harris, Sameer S Kadri

**Affiliations:** National Institutes of Health, Bethesda, Maryland; University of Maryland School of Medicine, Baltimore, Maryland; University of Maryland School of Medicine, Baltimore, Maryland; University of Maryland School of Medicine, Baltimore, Maryland; NIH - Critical Care Medicine Department, Bethesda, MD; Uniformed Services University of the Health Sciences, Bethesda, Maryland; University of Maryland School of Medicine, Baltimore, Maryland; National Institutes of Health Clinical Center, Bethesda, MD

## Abstract

**Background:**

Sepsis from hospital-acquired infection is associated with high mortality, is often caused by antimicrobial resistant (AMR) pathogens, and prompts the use of broader-spectrum empiric antibiotics. While this may ensure adequate coverage for some patients, many may receive excessively broad-spectrum antibiotics. This study evaluated the epidemiology of clinically suspected culture positive hospital-onset (HO) sepsis in 12 Maryland hospitals.

**Figure 1:**
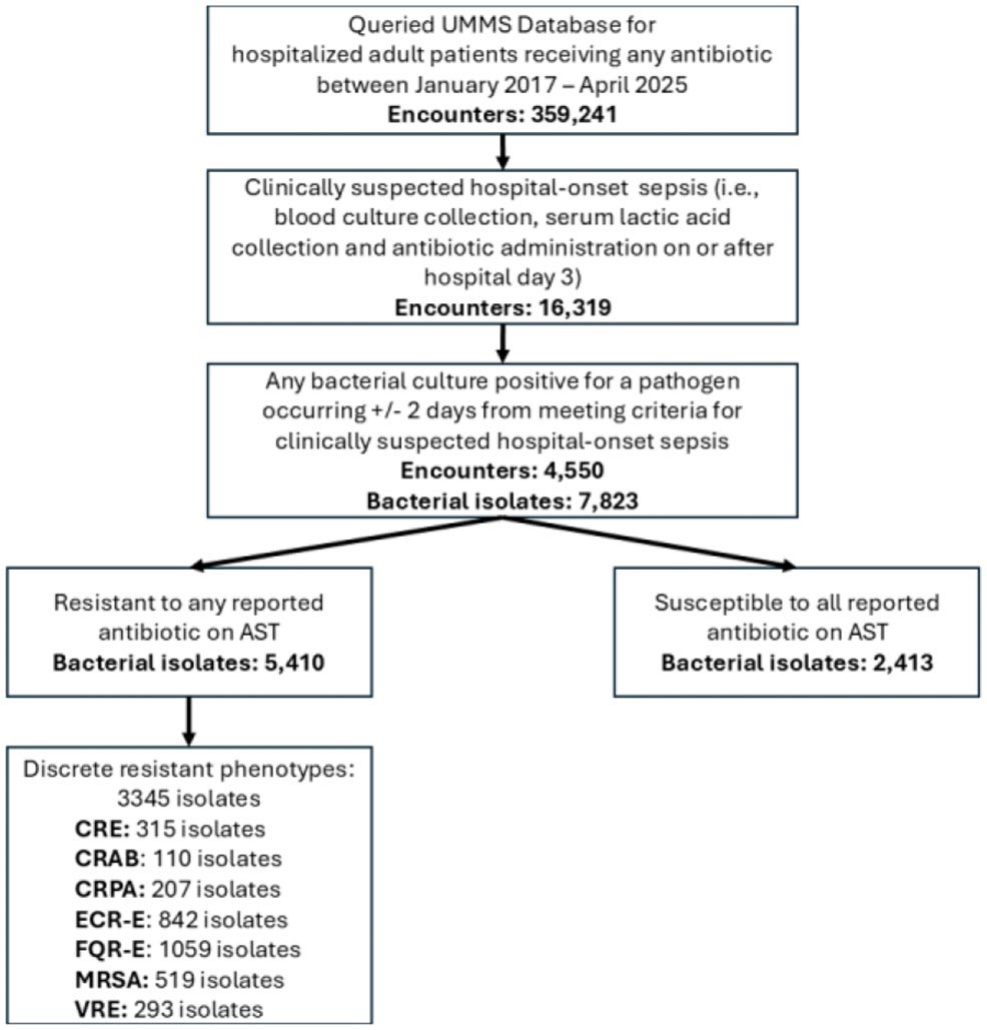
Patient Cohort CRE; carbapenem resistant Enterobacterales. CRAB; carbapenem resistant A. baumannii. CRPA; carbapenem resistant P. aeruginosa. ECR-E; extended spectrum cephalosporin Enterobacterales. FQR-E; fluoroquinolone resistant Enterobacterales. MRSA; methicillin-resistant S. aureus. VRE; vancomycin resistant Enterococcus.

**Figure 2:**
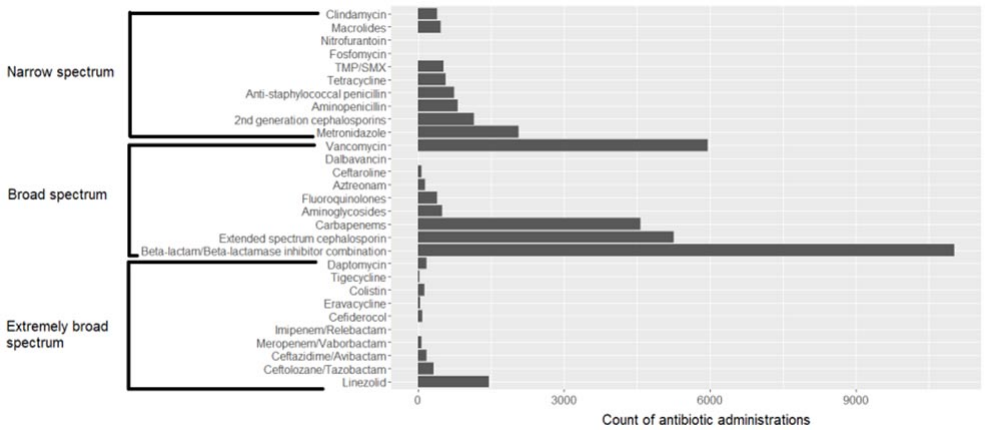
Distribution of antibiotics administered on the day a patient met criteria for culture positive clinically suspected hospital-onset sepsis. All antibiotic treatments administered on the day a patient met criteria for clinically suspected hospital-onset sepsis are represented. Antifungal therapies were excluded.

**Methods:**

We queried the University of Maryland Medical System database for adult encounters between Jan 2017-Apr 2025. Clinically suspected culture positive HO sepsis required simultaneous receipt of blood culture and serum lactate testing, and parenteral antibiotics on or after hospital day 3. Discrete resistant phenotypes were determined using available antimicrobial susceptibility testing (AST). Inadequate initial antibiotic therapy was defined as receipt of no *in vitro* active antibiotic and determined for patients with AST data corresponding to antibiotics administered on the day inclusion criteria were met. Broad-spectrum antibiotics were active against methicillin-resistant *S. aureus* and *P. aeruginosa* whereas extremely broad-spectrum antibiotics were active against vancomycin-resistant *Enterococcus* and carbapenem-resistant pathogens.

**Figure 3:**
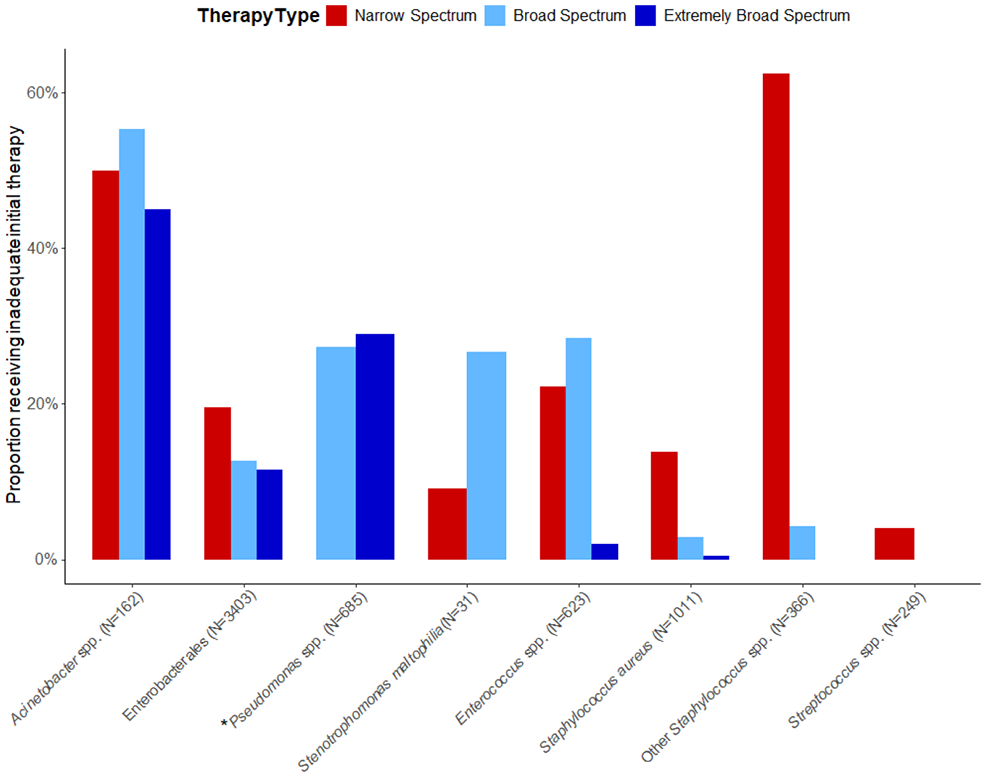
Proportion of isolates receiving inadequate initial antibiotic therapy across pathogen groups and stratified by spectrum of initial antibiotic therapy. Broad-spectrum antibiotics include those active against methicillin-resistant S. aureus and P. aeruginosa whereas extremely broad-spectrum antibiotics include those active against vancomycin-resistant Enterococcus and carbapenem-resistant pathogens. Narrow spectrum antibiotics include all others. Only isolates with antibiotic susceptibility testing data that correspond with the antibiotic administered on the day hospital-onset suspected sepsis criteria were met are represented (N=4120). Total N of unique isolates per pathogen reported in parenthesis. Multiple isolates were allowed per patient and isolates from patients receiving antibiotics from ≥1 antibiotic spectrum are represented in each respective bar. For example, a patient with an Acinetobacter spp isolate receiving both narrow and broad spectrum initial antibiotic therapy would be represented in each bar for this single isolate. *No narrow spectrum antibiotics have activity against Pseudomonas spp isolates and therefore no corresponding antibiotic susceptibility are reported.

**Figure 4:**
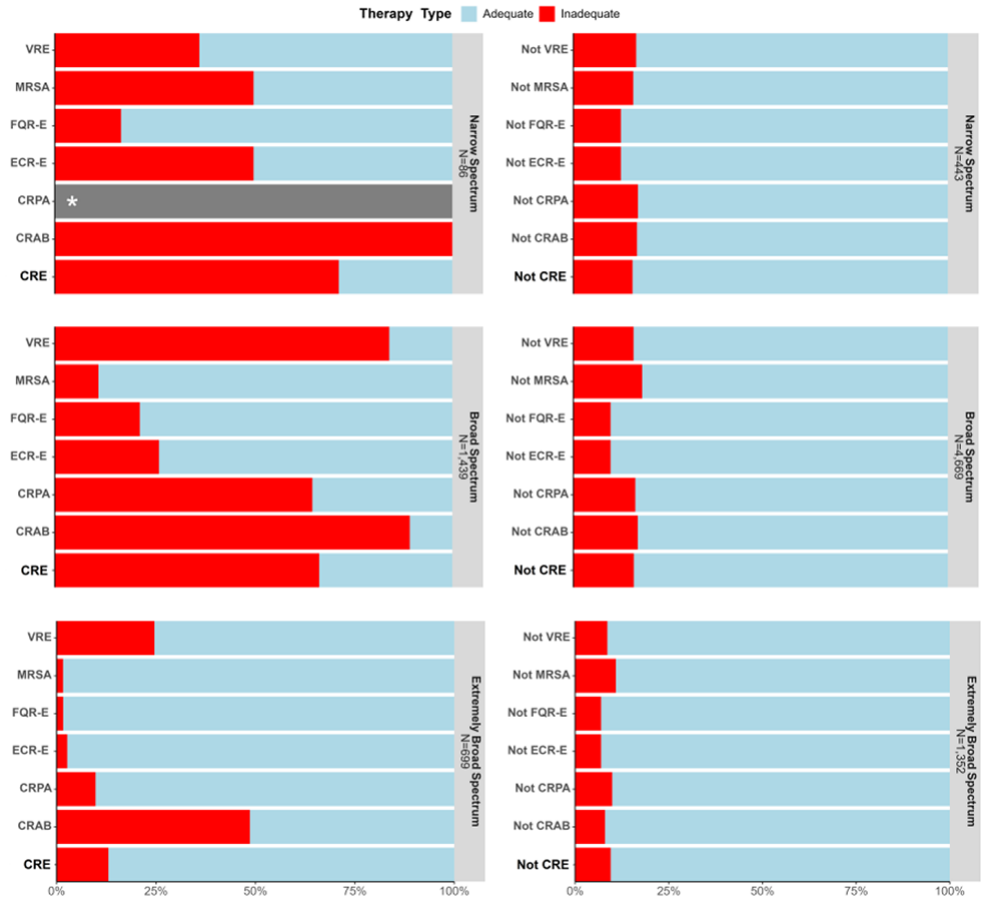
Distribution of inadequate and adequate initial antibiotic therapy stratified by discrete resistant phenotype and spectrum of initial antibiotic therapy. Broad-spectrum antibiotics include those active against methicillin-resistant S. aureus and P. aeruginosa whereas extremely broad-spectrum antibiotics include those active against vancomycin-resistant Enterococcus and carbapenem-resistant pathogens. Narrow spectrum antibiotics include all others. Only isolates with antibiotic susceptibility testing data corresponding to the antibiotic administered on the day hospital-onset suspected sepsis criteria were met are represented. *No narrow spectrum antibiotics have activity against Pseudomonas spp isolates and therefore no corresponding AST are reported. Total N of unique isolates per pathogen reported in parenthesis. Multiple isolates were allowed per patient and if a single isolate from a patient receiving antibiotics from ≥1 antibiotic spectrum then it is represented in each respective bar. CRE; carbapenem resistant Enterobacterales. CRAB; carbapenem resistant A. baumannii. CRPA; carbapenem resistant P. aeruginosa. ECR-E; extended spectrum cephalosporin Enterobacterales. FQR-E; fluoroquinolone resistant Enterobacterales. MRSA; methicillin-resistant S. aureus. VRE; vancomycin resistant Enterococcus.

**Results:**

Across 12 acute care hospitals, 4550 culture positive clinically suspected HO sepsis encounters were identified (Fig. 1). 31% (N=1393) of encounters died or were discharged to hospice. Among 7823 bacterial isolates across all encounters with interpretable AST results, 3345 (43%) displayed a discrete resistant phenotype. 18.2%, 75% and 6.8% of antibiotics administered as initial therapy were narrow, broad, and extremely broad-spectrum (Fig. 2). Even broad-spectrum empiric therapy was frequently inadequate across all pathogens and especially among resistant pathogens (Fig. 3-4).

**Conclusion:**

Unlike community-onset sepsis in US hospitals, AMR is frequent in HO sepsis and renders common broad-spectrum empiric antibiotics ineffective. Earlier identification of HO sepsis and resistant pathogens is a critical solution given the high lethality and the risk of *de novo* resistance development with indiscriminate empiric use of extremely broad-spectrum agents.

**Disclosures:**

Anthony Harris, MD, MPH, UpToDate Wolters Kluwer Health: Infection control section editor

